# Explanation and relations. How do general practitioners deal with patients with persistent medically unexplained symptoms: a focus group study

**DOI:** 10.1186/1471-2296-10-68

**Published:** 2009-09-24

**Authors:** Tim C olde Hartman, Lieke J Hassink-Franke, Peter L Lucassen, Karel P van Spaendonck, Chris van Weel

**Affiliations:** 1 Department of Primary and Community Care, Radboud University Nijmegen Medical Centre, Nijmegen, The Netherlands

## Abstract

**Background:**

Persistent presentation of medically unexplained symptoms (MUS) is troublesome for general practitioners (GPs) and causes pressure on the doctor-patient relationship. As a consequence, GPs face the problem of establishing an ongoing, preferably effective relationship with these patients. This study aims at exploring GPs' perceptions about explaining MUS to patients and about how relationships with these patients evolve over time in daily practice.

**Methods:**

A qualitative approach, interviewing a purposive sample of twenty-two Dutch GPs within five focus groups. Data were analyzed according to the principles of constant comparative analysis.

**Results:**

GPs recognise the importance of an adequate explanation of the diagnosis of MUS but often feel incapable of being able to explain it clearly to their patients. GPs therefore indicate that they try to reassure patients in non-specific ways, for example by telling patients that there is no disease, by using metaphors and by normalizing the symptoms. When patients keep returning with MUS, GPs report the importance of maintaining the doctor-patient relationship. GPs describe three different models to do this; mutual alliance characterized by ritual care (e.g. regular physical examination, regular doctor visits) with approval of the patient and the doctor, ambivalent alliance characterized by ritual care without approval of the doctor and non-alliance characterized by cutting off all reasons for encounter in which symptoms are not of somatic origin.

**Conclusion:**

GPs feel difficulties in explaining the symptoms. GPs report that, when patients keep presenting with MUS, they focus on maintaining the doctor-patient relationship by using ritual care. In this care they meticulously balance between maintaining a good doctor-patient relationship and the prevention of unintended consequences of unnecessary interventions.

## Background

In 25 to 50 percent of the contacts patients present medically unexplained symptoms (MUS) to the general practitioner (GP) [1,2]. Although it is only a minority (2.5 percent) of these contacts that will result in a chronic condition associated with recurrent consultations, extensive investigations and referrals, this minority of chronic patients represents a serious problem in primary care [3]. Persistent presentation of MUS is troublesome for the GP because many GPs experience difficulties in the communication and the relation with these patients [4-11]

There is evidence that the difficulties in communication may result from misperceptions of patients' needs and worries by GPs [12]. GPs feel pressurized by patients to apply biomedical interventions but they do not have much to offer in a strictly biomedical way [13,14]. At the same time, most patients with MUS do not overtly insist on additional somatic interventions. They primarily want to be understood and seek emotional support, which doctors do not provide [15-18]. Moreover, the lack of a biomedical explanation hinders GPs in adequately telling patients what is wrong [19]. As a consequence patients' needs are unmet, reassurance will be hampered and relief of symptoms will be complicated [20,21]

Recent research has shown that the current management of patients with MUS should consist of: communicating to the patients that the symptoms are real, making patients feel understood, engagement of the GP to establish common ground with the patient, offering a detailed explanation about the nature of the complaints, and if necessary symptomatic relief [22-25]

By definition, patients with persistent MUS will keep attending the consulting hours of their GPs with MUS. However, they will also face other health care problems, for example serious somatic disease [26]. Given the importance of a good relation with the patient in the light of continuity of care, GPs have to find strategies to specifically deal with these patients [10]. To our knowledge it is not known how GPs do this and how, according to GPs, this relationship evolves over time [27]. Furthermore, we need to know GPs' opinions about explaining the nature of unexplained symptoms to patients with persistent MUS during consultations, in order to develop more effective interventions for these patients.

In this qualitative exploratory study we focus on GPs' perceptions of giving explanations to patients with persistent MUS and on GPs' perceptions about the doctor-patient relationship and how the relationship evolves over time.

## Methods

We conducted five focus groups with 22 Dutch GPs to study their views on MUS. Each focus group consisted of 4 to 5 GPs. We used a purposive sampling strategy to increase the external validity of our results with respect to the variety of existing views among GPs. From the literature, we considered the following characteristics as relevant for this variety: age, gender, working experience, number of listed patients, 'academic working career', geographical location of practice (city versus rural) and site of education [28-31]

Each focus group was homogenous for the characteristics 'academic working career' and 'geographical location' (table 1); participants otherwise represented a variety of the listed characteristics (table 2).

**Table 1:** Focus group characteristics

	**characteristic**
Focus group 1	GPs with an academic working career in Radboud University Nijmegen Medical Center
Focus group 2	GPs without an academic working career working in a rural area
Focus group 3	GPs without an academic working career working in a rural area
Focus group 4	GPs with an academic working career in VUmc Amsterdam or Academic Medical Center University Amsterdam
Focus group 5	GPs without an academic working career working in a city

**Table 2:** Key characteristics of purposive sample of general practitioners

	**Number of general practitioners**
Gender	
Male	14
Female	8
Working hours	
Full time*	10
Part time	11
Not practicing at the moment	1
Type of practice	
Solo	1
Duo	2
Group	17
Variable	1
Not practicing at the moment	1
Urbanization	
Rural	3
Suburban	6
Urban	11
Variable	1
Not practicing at the moment	1
Age in years (range)	47 (31-58)
Experience as a GP in years (range)	15 (0-30)

* full time: 80% to 100% full time

We chose focus groups rather than individual interviews to use group interaction which stimulates participants to explore and clarify their views into more depth [32]. Discussions were facilitated by a skilled moderator, and lasted for approximately one and a half hour.

Following the guidelines for conducting focus groups, the moderator used a discussion guide to direct the discussion and to fulfil the research aims (table 3). The discussion guide was mainly based on important topics highlighted in the literature.

**Table 3:** Focus group interview guidebook

What are the characteristics of patients with persistent MUS?	- Regarding patient characteristics?
	- Regarding symptom characteristics?
	- Do you have problems to recognize these patients?
How do you call patients with persistent MUS?	- Which terms do you use to characterize these patients?
	- Which terms do you tell to your patients?

What's the aetiology of persistent MUS?	- What is the nature of these symptoms?
	- When do patients experience these symptoms?
	- Why do these symptoms persist for such a long time?

Do you explain the diagnosis persistent MUS to your patients?	- Do you think explanation is important in consultations with these patients?
	-How do you explain the diagnosis persistent MUS to the patient?
	-Which specific words do you use during explanation of the symptoms?

How do you manage patients with persistent MUS?	-How do you deliver health care to them?
	-What do you do with requests for additional research?
	-Which problems do you face in the management of these patients?
	-How do you manage diagnostic uncertainty?
	- Do you feel capable to manage these patients?

How do you describe the doctor-patient relationship with those patients?	-Is the doctor-patient relationship important, and why?
	-Do you experience problems in the doctor-patient relationship?

How do you experience the MUS consultations?	- Which problems do you face during the MUS consultation?

The discussions were tape-recorded with the participants' consent and completely verbatim transcribed. Data collection and analysis proceeded as an iterative process. Two researchers (ToH, LH) added relevant and new topics to the discussion guide after a preliminary analysis of each session. In this way, ideas and thoughts that emerged in primary stages of the analysis were brought forward in subsequent focus groups as the study proceeded.

Finally, the first author verified the transcription and entered all data into Atlas.ti, a software program used to support the analysis of qualitative data.

### Analysis

Analysis followed the principles of constant comparative analysis[33]. in which transcripts are subsequently thematically coded. The main aim of this analysis is to organize responses by theme and explore similarities and differences in and between groups.

Two researchers (ToH, LH) read all interviews several times to familiarize themselves with the data. They independently made a first categorization by coding meaningful sentences. Initial codes were discussed, seeking agreement on their content. In the event of disagreement, the opinion of a third researcher (PL) was sought. We grouped the codes into themes to identify key features of GPs' views on MUS. Recurrent and important themes were frequently discussed and refined as part of an ongoing iterative process [34]. During the entire analysis we constantly matched the developing themes with the transcripts and with available scientific literature on this subject. Therefore, these repeated themes are grounded in the data and not imposed onto the data by the researcher. We also checked our developing themes for inconsistencies with the transcripts.

Data collection continued until saturation was reached with no new major themes arising from analysis of the fifth focus group.

The validity of our findings were explored by checking our results in an independent group of GPs who had no specific interest in MUS. They judged the results to be consistent with their perceptions and experiences [35]

## Results

The GPs in this study considered the explanation of the nature of the symptoms as well as maintaining the doctor-patient relationship as a difficult but important task in helping patients with persistent MUS.

GPs with an academic working career discussed more about the classification and current theories about patients with persistent MUS. GPs without an academic working career had a more clear focus on the difficulties they experience in daily practice working with these patients. We could not find further major differences between the perspectives of academic and non-academic GPs. Furthermore, we could not find differences in perspective between rural and urban working GPs.

### GPs' perceptions of giving explanations

Importance of explanations and difficulties in explaining were recurrent themes in the focus group discussions. The first focus group discussion (GPs with an academic working career in Nijmegen) revealed that difficulties in a good explanation was an important topic in consultations with MUS patients. During the focus groups with GPs without an academic working career (focus group 2, 3 and 5) we discussed in depth the ways of explaining MUS to the patient. Both GPs with an academic working career (focus group 4) as GPs without an academic working career stressed the importance of a clear explanation. In focus group 5, no new themes on the importance of explanation came up.

GPs stressed the importance of a clear explanation of the symptoms. An adequate explanation was regarded as important in both reassuring patients that there is no serious disease, and in helping patients to accept that there is not always a medical explanation for physical symptoms.

"GP13 (male, 5 years GP working experience): But you need to explain damned well. GP12 (male, 29 years GP working experience): The doctor has the monopoly of truth, so you need to be very clear about the cause of the symptoms. Don't be vague because otherwise a patient will return home muddled which make things worse [FG 3]

GPs stated that adequate formulated explanations may help patients understand the connection between their psychosocial life and the symptoms. According to the GPs, patients' family members and patients' colleagues also wish an explanation of the symptoms too, especially when patients have benefits of being ill.

Although GPs firmly agreed on the importance of a clear explanation of the symptoms they experience difficulties in doing this. GPs have difficulties indicating from which conditions the symptoms originate. We see this from the vague and avoiding answers of the GPs to the questions of the moderator and the long silence after a question of the moderator on this topic.

"GP4 (male, 26 years GP working experience): I explain to patients which symptoms are bothering them and I leave the diagnosis in the middle. I accept the symptoms as such and ask about the consequences of the symptoms. GP3 (female, 8 years GP working experience): Yes, I avoid diagnostic terms too and I confine myself to the particular symptom and I explain that it could be anything. Moderator: But which terms do you use? (moments of silence) GP2 (male, 17 years GP working experience): I discuss with them a different way of coping with their symptoms which may relieve them." [FG 1]

"GP5 (male, 7 years GP working experience): In these patients there is often no connection between symptoms and problems in daily life. At least I can't see one. GP7 (male, 25 years GP working experience): I often tell them that we are not yet knowledgeable. Particularly about patients who really have difficulties with their symptoms, yes, you have to respond differently. GP5: But, when you ask me 'where exactly do the symptoms come from, that chronic fatigue', then I don't have an answer" [FG 2]

Our analysis revealed three approaches, according to GPs, to explain the unexplained symptoms.

First, GPs indicate that they tell patients there is no disease. However, GPs highlighted the dilemma of how to communicate the finding of a patient suffering from symptoms without evidence of any physical anomaly. They describe that they try to reassure patients with statements that 'nothing is wrong'.

"GP9 (female, 19 years GP working experience): Yes, I always say: I don't know it either. It is not your heart, not your lungs, we did not find any abnormality. GP6 (female, 1 year GP working experience): Yes, I recognize what [GP9] is saying: at least we can conclude that it's nothing serious. We have examined a lot, but the question as to what you are actually suffering from is difficult to answer. GP5 (male, 7 years GP working experience): Yes, these symptoms are not caused by a disease" [FG 2]

Secondly, GPs indicate that they *use metaphors *to give patients some insight in the hypothesized interactions between symptoms and psychosocial life. GPs reported that they use metaphors - often a tangible physical mechanism indicating some kind of imbalance between load and capacity - that reflect their tacit beliefs and ideas about the nature of MUS. According to the GPs, sometimes the metaphor facilitates a discussion of psychological or social problems.

"GP5 (male, 7 years GP working experience): Every human being has a weak spot and if there's something wrong you feel it there. GP9 (female, 19 years GP working experience): I always tell them to compare it with a heavily overloaded elevator. GP7 (male, 25 years GP working experience): I recognize your story, I always tell patients that everyone has a backpack and this backpack can be too heavy." [FG 2]

Thirdly, GPs indicate that they *normalize the symptoms *of the patients, telling patients that having symptoms is a part of normal life. GPs reported that they explain to the patient that the symptoms are within a common, acceptable range, that they are not dangerous and that diagnostic procedures or treatment are not necessary.

"GP10 (female, 1 year GP working experience): I normalize. I mean, I explain to patients that it is normal, that it's not strange. I try to normalize as much as possible. GP12 (male, 29 years GP working experience): Yes. GP10: saying that it is part of normal life. GP12: Yes" [FG 3]

### GPs' perceptions of the evolving doctor-patient relationship

In all focus groups GPs discussed the importance of the doctor-patient relationship. The difficulties arising in the relationship was a recurrent theme. Focus groups with GPs without an academic working career (focus group 2, 3 and 5) discussed the way of dealing with the doctor-patient relationship in patients with persistent MUS. Furthermore, focus group 4 and 5 GPs discussed the difficulties they face in the relationship with these patients.

GPs intend to clarify the link (as supposed by them) between somatic experiences and psychosocial circumstances of the patient. In other words, they indicate to try to *change the agenda*. When talking about psychosocial circumstances is not successful, GPs reported that they suggest and discuss a range of activities: doing some sports, giving more frequent consultations for the symptoms, using a symptom diary, taking medication or referring to a social worker.

When changing the agenda doesn't work out well, GPs reported that they focus on *dealing with the doctor-patient relationship*. Within this strategy we could distinguish three different doctor-patient relationship models.

A first doctor-patient relationship model can be characterized as *mutual alliance*. This alliance is realized by some sort of ritual care. GPs stated that they use rituals with seemingly mutual approval and that these rituals emerge gradually after many consultations.

"GP9 (female, 19 years GP working experience): At a certain moment in your approach of the patient, when a patient has had all diagnostic procedures and many referrals, then comes the moment when one realizes: this is the only way this patient can live. Consequently I let him consult me sporadically, even without complaints [...] And when he feels such a ritual is sufficient, examining his hart, lungs and blood pressure, reassurance is reached to keep him happy for some time. [...] GP7 (male, 25 years GP working experience): Finally, you have created some kind of relationship, some kind of game in which patients are quite satisfied with little. Someone listening [...] a pat on the back." [FG2]

Examples of those rituals are regular physical examination, referral to a physiotherapist, prescribing medication or performing additional investigations, all with preserving a good relationship with the patient and keeping in mind the unintended consequences of unnecessary interventions.

GP16 (male, 23 years GP working experience): Sometimes I just wait and see, and take care not to cause any damage in these patients" [FG4]

GPs said that these rituals are connected with requests or wishes of the patients and that they primarily aim at reaching agreement. GPs reported that they provide this kind of care with warmth and empathy and that they assume that patients are satisfied with it.

The second doctor-patient relationship model is *ambivalent alliance*. As stated by the GPs, this model is characterized by the same rituals as in the first model, but in fact the GPs do not agree with the rituals and are unhappy with the situation. There is often a negative colouring in GPs' utterances about this method.

"GP14 (male, 15 years GP working experience): Nowadays I ask patients to undress and I practice all sorts of complicated physical examinations I can think of, and when I have done all the physical examinations; then I say: everything is all right, you are healthy and then they go home satisfied [...] A patient visits me six times for a referral note, yes, when he or she comes for the third time, then I agree with a referral, inevitable, otherwise you have an argument and in my working experience that does not work at all [...] [FG4]

GPs reported that the ambivalent alliance indicates a disagreement with supposed requests for medical interventions as medical necessity of these interventions is doubtful. They reported that in these situations the patient is in control of the situation.

The third relationship model appearing from the discussions is *non-alliance*. GPs reported that this model rarely occurs in daily practice. They stated that this model is characterized by cutting off all reasons for encounter in which symptoms are not of somatic origin by taking a cool, objectifying medical gaze.

"GP12 (male, 29 years GP working experience): sometimes it is easier to be very short, in a way of 'you have to find out for yourself'. Go to a social worker and do not bother me with that problem again. Just be practical. Then it does not bother me at all. The patient is the one with the problem, it is not my problem, it is your problem, and you have to solve that with the social worker." [FG3]

In this non-alliance model, GPs reported that in case of an absence of a somatic explanation for the symptoms, they communicate this negative finding directly to the patient and at the same time give the message that the patient should not consult with these kinds of problems.

Analysis in and between the focus group discussions revealed that each GP has a preferred way of handling the relationship problem. GPs who stressed the importance of the relationship preferred the mutual alliance model, whereas GPs who stressed the importance of changing the agenda seemed to rely on the ambivalent alliance model. Non-alliance was not frequently mentioned. One GP stated that he coped with patients with persistent MUS in such a way during his GP residency. One GP told that he used this non-alliance model to cope with these patients during out-of-our-services.

## Discussion

GPs are aware of the importance of explaining the diagnosis of MUS adequately to patients with persistent unexplained symptoms. However, they face difficulties in explaining the nature of the symptoms during the encounter with these patients. GPs state that they use three different approaches to explain the symptoms to the patients; normalization of symptoms, telling patients that there is no disease, and using metaphors. According to the literature, normalization of symptoms and telling patients that they don't have a disease is not effective and may even result in more health-care seeking [19,36]. This might contribute to the fact that a small but relevant proportion of MUS patients become persistently impaired and keep attending the GP [37]. Metaphors, on the other hand, can be useful in reaching shared understanding between patient and doctor because they are tangible and non-blaming, although there is limited evidence for their effectiveness [36,38]. Seemingly, there is a paradox in arguing that physicians should provide explanations for a problem that they themselves describe as unexplained. However, most complaints presented in primary care remain at the level of a symptom diagnosis and never result in the diagnosis of a disease [39]. The connotation 'unexplained' in medically unexplained symptoms indicates that the symptoms are not explainable from the reductionist disease framework [40]. However, these symptoms are frequently explainable in other terms given by models as the somatosensory amplification model or the cognitive-perceptual model [41]. Apparently, GPs lack the competence to use these available models adequately in patients presenting with persistent MUS. However, searching for a symptom explanation together with the patient is an important task of GPs in daily practice as it gives them the opportunity to establish common ground on which they can jointly understand and manage the patients' needs [42]

GPs realize the usefulness and importance of a good relation in encounters with these patients, although they face difficulties in putting this into practice when explaining and removal of symptoms is not feasible. When patients keep presenting MUS, GPs report that the doctor-patient relationship evolves into three different models characterized by the presence or absence of mutual understanding and a careful balance between maintaining the doctor-patient relationship and preventing unintended consequences of the interventions.

Although GPs recognize the limitations and difficulties of establishing an ongoing and preferably effective relationship with these persistent MUS patients in daily practice, they seems to take responsibility to build and maintain such a relationship. Taking this responsibility fits into the philosophy of primary care in which a long-term and continuous relationship in general practice is emphasized [20,43]. These relationships evolve over time and are built on regular consultations as well as other shared experiences [20,26]. It is known from the literature that building on and establishing an effective and satisfactory doctor-patient relationship has an appreciable impact on health outcomes for patients [44]

GPs stated that different relationship models with patients with persistent MUS develop over time: mutual alliance, ambivalent alliance and non-alliance. These relationship models are, as far as we know, not described elsewhere. The *mutual alliance *model, and to a lesser degree the *ambivalent alliance *model, can be conceptualized as comprising a positive relationship and collaboration with mutual approval between patient and doctor [45]. The goal of this collaboration is to maintain the doctor-patient relationship by providing emotional support through some kind of ritual care. In this strategy GPs keep in balance the doctor-patient relationship and the unintended consequences of interventions. Reaching mutual alliance corresponds with findings that patients with MUS seek a high level of emotional support rather than somatic interventions [17,46]

Chew-Graham pointed out that GPs who experience difficulties in the relationships with some groups of patients felt that concentrating on maintaining the doctor-patient relationship make them to collude with patients and their symptoms [47]. It is possible that GPs in the *'ambivalent alliance' *model are hindered by this collusion and feel unhappy with the ritual care.

One could argue that the presented models for the doctor-patient relationship are doctor-centred and the patient perspective is lacking. However, this may be the result of the aim of our study to examine GPs' perceptions. We asked the GPs in the focus groups how they manage patients with persistent MUS. In other words, we asked for their own GP perspective. In response they described how they struggle to preserve their relation with the patient. In this respect the GPs are working patient-centred. Moreover, the mutual alliance strategy as well as the ambivalent alliance strategy incorporate certain elements of patient-centredness such as finding common ground regarding management and enhancing the doctor-patient relationship [48]. GPs did not introduce several other aspects of patient-centredness such as disclosing patients' concerns and suffering, and focussing on patients' self management and coping [49]. However, this can be explained by the fact that during our focus group interviews we focussed on situations in daily practice in which GPs felt that they get stuck with these patient. Possibly, strategies as disclosing concerns, relief of suffering, and focussing on self management and coping had failed in an earlier stage of the doctor-patient contact.

### Strengths and limitations of this study

The qualitative method is appropriate to explore and clarify what GPs think about these patients and what they experience in the consultations with these patients [32]. However this method does not provide insight in the GPs actual behaviour. By using a cyclical and interactive way of collecting and analyzing data, 'progressive focusing' and exploration of GPs' perceptions in depth were realized [50]. In focus group discussions the participants influence each other by listening and discussing. Such group dynamics may silence individual contrasting opinions and result in the articulation of group norms or early consensus before all views were fully expressed [32]. However, the goal is not to reach consensus. Instead, our goal was to study how GPs as professionals think about patients with persistent MUS. It would nevertheless be interesting to analyze how individual participants influenced each other during the discussion, but this was not the aim of our study. To reach an optimal variety of opinions, we used a purposive sampling strategy. Although small, the number of participants is considered adequate for this purpose [32]. By using a purposive sampling strategy we have captured the variety of opinions present in the population of GPs.

As MUS are not equally distributed among men and women and men and women have different expectations and experiences of clinical encounters[6], a gender perspective may enhance understanding. However, we did not study the differences in thinking about patients with persistent MUS between female versus male GPs, as in this study we focussed on eliciting GPs' perceptions of giving explanations and their perceptions about the doctor-patient relationship.

Although we know from recent research that there are cultural differences in the distribution of MUS and the meaning and significance of a symptom depends on the perceived relationship with diseases in a culture [51-53], GPs did not spontaneously introduce their opinions on cultural aspects of persistent MUS during the focus group discussions.

This qualitative study examines GPs' perceptions and not actual behaviour. We deliberately chose to study the perceptions because actual behaviour may result from perceptions to a certain degree. Moreover, this study is part of a larger project in which we examine actual behaviour of GPs in a video registration study and the patient perspective in a qualitative interview study.

Tape-recording the discussion, multiple coding during analysis and our triangulation strategy of asking independent GPs to judge our results to be consistent with their own perceptions and experience, added to the rigor of the study.

Further studies using a mixed method methodology may reveal effective methods of explaining symptoms to patients with MUS. Moreover, it would be useful to study the effects of the three relationship models reported by the GPs on outcomes and satisfaction in patients. In research, as well as education we should face the challenge of explaining the unexplained symptoms and building a truly effective doctor-patient relationship with these fascinating patients. With the results of further research we would like to address the challenge of explaining unexplained symptoms adequately and building effective doctor-patient relationships with these patients, preferably by educating doctors with tools how to do so.

## Conclusion

GPs are aware of the importance of explaining MUS adequately to their patients, however they have difficulties in doing so. GPs report that, when patients keep presenting with MUS, they focus on maintaining the doctor-patient relationship by using ritual care. These relationships evolves into three different models characterized by the presence or absence of mutual understanding and a careful balance between maintaining the doctor-patient relationship and preventing unintended consequences of the interventions.

## Competing interests

The authors declare that they have no competing interests.

## Authors' contributions

All authors participated in the research process (study design: ToH, PL, CvW; data collection: ToH, KvS; data analysis and interpretation: ToH, LH, PL, KvS. ToH, LH and PL drafted the manuscript and all authors helped with revisions to the manuscript. All authors approved the final version.

## Funding

This study was supported by grant 920-03-339 from ZonMw (Netherlands Organization for Health Research and Development).

## Pre-publication history

The pre-publication history for this paper can be accessed here:


